# Gene transcription and chromatin regulation in hypoxia

**DOI:** 10.1042/BST20191106

**Published:** 2020-05-05

**Authors:** Michael Batie, Sonia Rocha

**Affiliations:** Department of Biochemistry, Institute of Integrative Biology, University of Liverpool, Biosciences Building, Crown Street, Liverpool L69 7ZB, U.K.

**Keywords:** chromatin, hypoxia, hypoxia-inducible factors, transcription

## Abstract

Oxygen sensing is an essential feature of metazoan biology and reductions in oxygen availability (hypoxia) have both physiological and pathophysiological implications. Co-ordinated mechanisms have evolved for sensing and responding to hypoxia, which involve diverse biological outputs, with the main aim of restoring oxygen homeostasis. This includes a dynamic gene transcriptional response, the central drivers of which are the hypoxia-inducible factor (HIF) family of transcription factors. HIFs are regulated in an oxygen-dependent manner and while their role in hypoxia is well established, it is apparent that other key players are required for gene expression control in hypoxia. In this review, we highlight the current understanding of the known and potential molecular mechanisms underpinning gene transcriptional responses to hypoxia in mammals, with a focus on oxygen-dependent effects on chromatin structure.

## Introduction

Molecular oxygen is a co-factor in various biochemical reactions and is essential for many aerobic organisms in their maintenance of intracellular ATP levels [1]. Responses to low oxygen (hypoxia) are highly conserved and vital to function and survival [2]. Dysregulated hypoxia response systems can lead to developmental issues as well as diseases [3], a notable example of which is renal clear cell carcinoma [4]. Hypoxia can occur in the context of a whole organism, for example during embryonic development where it is an important physiological cue, or when exposed to high altitude environments [3]. Hypoxia can also occur at the cellular level, when oxygen supply is reduced and/or metabolic demand is increased, for example in tumour hypoxia [5]. Importantly, normal physiological oxygen levels and the oxygen concentration at which hypoxia responses are activated are tissue and developmental stage specific.

The core aim of the hypoxia response is to restore oxygen homeostasis. To achieve this, a co-ordinated response involving changes in the regulation of gene expression, and protein stability and function are triggered, which impinges on many aspects of energy-related processes [6]. Given the importance of hypoxia to both normal and disease biology, understanding the mechanisms involved in oxygen sensing and adaptions is an attractive challenge to researchers.

A major breakthrough in our understanding of the hypoxia response came through the discovery of the von Hippel–Lindau tumour suppressor (VHL)-prolyl hydroxylase (PHD)-hypoxia inducible factor (HIF) pathway [2,7]. The HIF transcription factors are the primary mediators of hypoxia-induced gene transcriptional responses but do not act alone. There are additional factors that control HIF transcriptional activities and non-HIF dependent gene regulation in response to low oxygen, including regulators of the chromatin environment [8–11].

## Hypoxia inducible factor (HIF)

**Figure 1.**
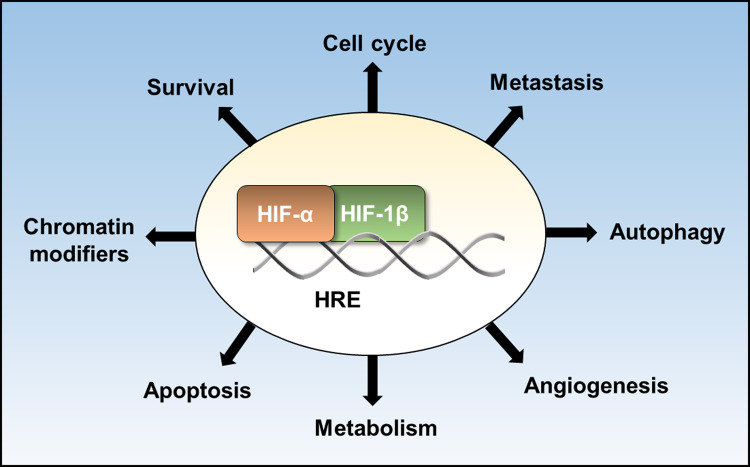
HIF pathways. Some of the biological processes regulated by hypoxia-inducible factor (HIF) target genes.

The HIF transcription factor family consists of oxygen labile α subunits, of which there are three homologues in vertebrates, HIF-1α, HIF-2α and HIF-3α, and a β subunit, HIF-1β. HIFs are the master regulator of the transcriptional response to hypoxia and bind to hypoxia response elements (5-(A/G)CGTG-3), typically functioning as transactivators [12,13]. To date, there are over 100 validated, direct HIF target genes, which are involved in biological processes (Figure 1). Canonical regulation of HIF-α subunits is via proline hydroxylation in their oxygen-dependent degradation domain by PHDs, which are 2-oxoglutarate (2-OG) dependent dioxygenases, requiring oxygen, iron and 2-OG for their catalytic activity [2]. Hydroxylated HIF-α provides a high affinity-binding site for the VHL E3 ubiquitin ligase complex. This leads to proteasomal degradation of HIF-α. PHDs can act as molecular oxygen sensors and, in hypoxia PHDs ability to hydroxylate HIF-α is impaired, enabling stable HIF-α subunits to translocate to the nucleus and dimerise typically with HIF-1β. Another HIF hydroxylase, factor inhibiting HIF1 (FIH), hydroxyxlates an asparagine residue is the C-terminal transactivation domain (CTAD) of HIF-1α and HIF-2α, inhibiting interaction with the co-activator CREB-binding protein (CBP)/p300 [14,15]. Similar to PHDs, FIH is a 2-OG dependent dioxygenase, and inhibition of FIH activity in hypoxia alleviates blockage of CTAD co-activator interactions [16]. Although controversial, there is an expanding list of non-HIF putative hydroxylation targets of PHDs and FIH, providing additional mechanisms by which they can influence the hypoxia response [17–19]. There are additional post-translation modifications of HIF subunits that regulate their stability and function [11], and control of HIF subunit transcription is also pertinent to HIF activity and responses to low oxygen [20].

There is a high degree of tissue-specific HIF transcriptional responses and the numbers of estimated HIF regulated genes can vary massively depending on the study and cell types analysed. Whilst in some cases, technological differences in approaches used may account for this, evidence points towards altered isoform expression and activity, and chromatin accessibility and local chromatin environment, including RNA pol II availability around HREs, as being a major determinant of cell-type specificity [21–27]. Chromatin regulation is likely also important in controlling HIF independent gene transcriptional responses to hypoxia and efforts are ongoing to determine the role of chromatin in hypoxia.

## Chromatin and hypoxia

Reduced oxygen can alter chromatin post-translational modifications, both directly via inhibition of oxygen-dependent enzymes and indirectly through additional mechanisms such as increased expression and/or HIF recruitment to chromatin [8,10,11,28–33].

Eukaryotic DNA is stored in the nucleus in the form of chromatin, consisting of DNA wrapped around histone octamers to form nucleosomes, which are linked together by linker DNA and histones and packaged into higher-order structures [34]. DNA replication, DNA repair and gene transcription are dependent upon the chromatin environment. Furthermore, chromatin can act as an accessibility barrier for gene transcription [35,36]. A closed chromatin conformation with nucleosomes tightly packaged can block the accessibility of transcriptional regulators and machinery to target genomic loci, silencing gene transcription. Alternatively, a more open conformation at gene regulatory regions is permissive to the binding of transcriptional regulators and machinery.

Mechanisms utilised by cells to modulate chromatin structure include, chromatin remodelling by ATP dependent chromatin remodellers [37], incorporation of histone variants [38], action of non-coding RNAs [39], DNA methylation [40] and histone post-translational modifications [41]. These mechanisms of chromatin modulation combine to dictate gene transcriptional output and cell fate decisions.

The impact of low-oxygen signalling on the chromatin environment, focusing on chromatin post-translational modification and chromatin organisation will be discussed in this review. Roles chromatin remodeller complexes, non-coding RNAs and incorporation of histone variants in hypoxia are also important factors but will not be detailed here.

## Histone methylation

**Figure 2.**
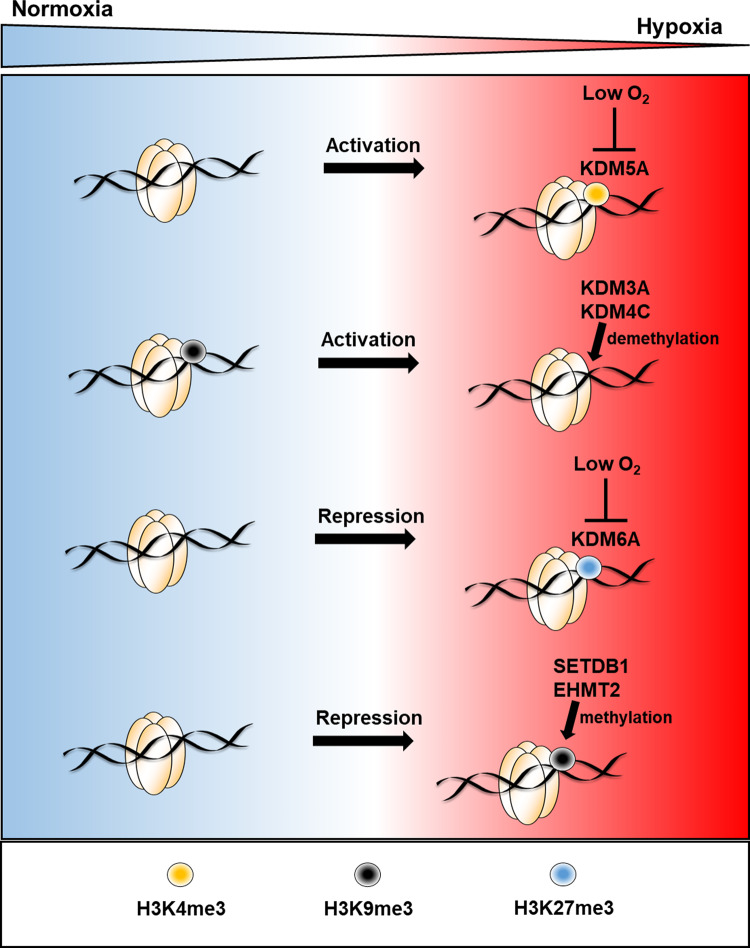
Histone methylation mediated regulation of gene transcription in hypoxia. Hypoxia-induced changes in histone methylation, their effect on gene expression and the enzymes directing these changes. Lysine-specific demethylase (KDM), SET domain bifurcated 1 (SETDB1), euchromatic histone lysine methyltransferase 2 (EHMT2).

Histone methylation is a reversible modification to lysine residues of histone N-terminal tails. Some histone methylation modifications are associated with active gene transcription such as H3K4me3 and H3K36me3 whereas as others, such as H3K9me3 and H3K27me3 are associated with gene silencing [41]. The major family of histone demethylases, which remove methyl groups from histones lysine residues are the Jumonji-C (JmjC) proteins [42]. There are 32 JmjC proteins which have been shown to demethylate histones in humans and are classified into subgroups based on sequence homology and histone substrates (lysine specific demethylase, KDM2–8) [8]. JmjC proteins, like the HIF hydroxylases, PHDs and FIH, are 2-OG dependent dioxygenases. Several groups have reported hypoxia causes increases in total levels of various histone methylations; some have attributed this to inhibition of JmjC protein activity [29,30,33,43–49]. Recently, Batie et al. [29] and Chakraborty et al. [30] provide evidence that oxygen sensing by JmjC proteins in hypoxia results in increased levels of certain histone methylations, which co-ordinates the transcriptional response to hypoxia (Figure 2). It is clear from both *in vitro* and cell culture studies that different JmjC proteins have different oxygen sensitivities and this combined with their relative expression will contribute to their activity in oxygen-deprived environments. Many JmjC proteins are hypoxia induced, with some being HIF targets [8,50]. KDM4C and KDM3A can also function as co-activators of hypoxia-inducible gene expression in hypoxia via H3K9me3 demethylation, facilitating gene transcriptional activation [51,52] (Figure 2). Increased expression of JmjC proteins may in part act as a feedback mechanism to help retain activity, as is seen with PHD2 and PHD3.

SET domain bifurcated 1 (SETDB1) and euchromatic histone lysine methyltransferase 2 (EHMT2) are H3K9 targeting histone methyltransferases (HMTs) that have been found to mediate gene repression in hypoxia via H3K9 methylation (Figure 2), with implications hypoxia-induced p53-dependent apoptosis and tumour growth, respectively [53,54]. Increased H3K9me3 in hypoxia is also required for A-T mutated (ATM) activation via epigenetic silencing of ATM-specific phosphatases, which facilitates DNA replication in a low-oxygen environment [47].

Direct oxygen sensing may also affect DNA methylation. DNA methylation is associated with gene silencing and ten-eleven translocation (TET) enzymes, which are involved in DNA demethylation, are also 2-OG dependent dioxygenases. It has recently been shown that TET enzyme inhibition in hypoxia increases global levels of DNA methylation, thus oxygen sensing via TETs in hypoxia may also contribute to transcriptional regulation [31]. Hypoxia-induced DNA hypermethylation has also been attributed to HIF-1α and transcriptional up-regulation of methionine adenosyltransferase 2A (MAT2A) [55].

## Histone acetylation

Histone acetylation is associated with transcriptional activation [41]. It involves the transfer of an acetyl group from acetyl CoA to certain lysine residues on side chains within the positively charged N-terminal tail region of histones. This is a reversible histone modification, with acetyl group addition catalysed by histone acetyl transferases (HATs) and removed catalysed by histone deacetylases (HDACs). Acetylation neutralises the positive charge at histone tails and increases local chromatin accessibility. Whereas removal of acetyl groups is typically associated with increased chromatin compaction and localised transcriptional repression. Histone acetylation can also function as a binding site for chromatin-associated proteins containing a bromodomain, such as chromatin remodellers [37].

HATs and HDACs play a complex and dynamic role in the regulation of gene transcription in hypoxia via a multitude of mechanisms [9]. Focusing on direct regulation of gene expression through histone acetylation/deacetylation functions, lysine acetyltransferase 5 (KAT5) (also known as TIP60) and CBP/p300 are known HIF co-activators which interact with HIF, acetylate histones at HIF bound loci and are required for transcriptional activation at a subsets of HIF target genes [11,56]. Whereas HDAC1 interacts with HIF and reduces gene transcription at HIF targets via histone deacetylation [57]. SIN3 transcription regulator family member A (SIN3A) forms co-repressor complexes with HDACs and Tiana et al. [58] found that SIN3A binding is enriched both at hypoxia induced and repressed genes in human endothelial cells. SIN3A was required for down-regulation of 75% of the hypoxia-repressed genes and for the up-regulation of 47% of hypoxia-induced genes. In this study, SIN3A binding was unaffected by hypoxia, but H3K27Ac levels correlated with gene transcription, suggesting that HDAC activity at SIN3A complexes in hypoxia is a key factor in how SIN3As effects gene transcription. Detailed analysis of local chromatin accessibility in response to altered oxygen availability is lacking, as is the contribution of HATs and HDACs through histone acetylation/deacetylation. Johnson et al. [44] found that promoter nucleosome occupancy is altered in hypoxia at small group of hypoxia-responsive genes, with levels of nucleosome occupancy inversely correlating with hypoxia-induced gene expression. More recently, Suzuki et al. [59] identified hypoxia-inducible, nucleosome-free regions within the promoters of some hypoxia-inducible genes. These changes were HIF dependent and reversible upon reoxygenation. Interestingly, nucleosome reassembly through reoxygenation required SIN3A but not HDAC classes I and II activity.

Although limited, there are reports of hypoxia effecting total levels of acetylated histones. Decreased H3K9Ac [43,44] and H3K27Ac [60] and increased H3K14Ac [44] has been shown in cell culture models of hypoxia. Mechanistic insight into these changes and the extent to which they are driven by altered HAT/HDAC enzymatic activity or differential chromatin recruitment remains unclear.

## 3D chromatin organisation

Eukaryotic genomes inside the nucleus require a complex three-dimensional organisation. This organisation includes chromatin loops and topological associating chromatin domains, which modulate gene transcription [61]. Sequencing approaches employed by various groups have increased our understanding of HIF mediated transcriptional regulation on genome-wide scale, providing some insights into chromatin arrangement. Through the use of RNA-seq, ChIP-seq, DNAse-seq, GRO-seq and Capture-C, researchers find that HIF functions mainly by binding to permissive HRE containing regulatory elements with pre-bound and paused RNApol II [21–27,33,62,63]. HIF binding and coactivation subsequently triggers the release of paused RNA pol II at the majority of targets. It is now appreciated that HIFs bind to promoter distal, as well as, proximal regulatory elements, and that promoter enhancer interactions through chromatin looping is important in the HIF response. Work from Mole and Ratcliffe laboratories, using Capture-C and ChIP-seq, mapped chromatin–chromatin interactions at a subset of HIF target promoters [27]. This study found that HIF promoter distal binding occurs at pre-established and primed, promoter enhancer loops in VHL reconstituted 786-O cells and MCF7 cells. Using DNase-seq, Choudhry et al. [26] demonstrated that the average accessibility signal at proximal promoters of the top 100 hypoxia-inducible genes determined by RNAs-seq was unchanged in MCF-7 cells treated to 1% oxygen for 24 h 26].

Despite these insights, there is limited information regarding the effects of hypoxia on chromatin organisation in hypoxia, particularly in an unbiased manner. Using single-molecule localisation microscopy (SMLM) and *in situ* DNA digestion coupled with fluorescent microscopy, Kirmes et al. [64] observed a rapid change in chromatin architecture and elevated chromatin compaction in cardiomyocytes deprived of oxygen and nutrients. Furthermore, total transcription rates were reduced and chromatin and transcription changes where rapidly reversed upon restoration of oxygen and nutrients. Mechanistically changes in chromatin architecture were linked to reduced intracellular ATP and elevated polyamines. We can speculate that this chromatin conformational change in ischaemic conditions is strategic for the conservation of cellular energy. This is also occurring for the process of translation in severe hypoxia and ischaemia, with observed reduced translational rates in cells [65]. Reduced sensitivity to Mononuclease digestion, indicative of increased heterochromatin and increased global chromatin compaction, and increased levels of heterochromatin protein 1 binding protein 3 (HP1BP3), a heterochromatin structural protein, was determined in A431 cells exposed to severe hypoxia [66]. Therefore, HP1BP3 may be involved in hypoxia-induced chromatin compaction. Given that some chromatin-modifying proteins utilise oxygen and metabolites, chromatin may directly and dynamically sense changes in oxygen and metabolites during hypoxia, resulting in changes to chromatin organisation.

## Summary

There are still many questions concerning how different cell and tissue responses to low-oxygen environments are determined. There is growing evidence for a dynamic role of chromatin in sensing and responding to hypoxia to facilitate transcriptional changes, both via and independent of HIF. This includes changes in histone and DNA modification and localised chromatin compaction. Further elucidating the complex, co-ordinated mechanisms of chromatin regulation in low oxygen will be essential to better understanding hypoxia in normal physiological responses and disease.

## Perspectives

- Sensing and responding to changes in oxygen availability is paramount for organismal and cellular survival. Changes in gene expression are at the forefront of cellular responses to hypoxia, with the transcription factor family hypoxia inducible factor (HIF) as a master regulator.
- Very recently, chromatin epigenetic marks have been shown to be essential for gene expression changes in hypoxia as well as cellular fate.
- Over the coming years, the use of advanced techniques such as chromatin conformation capture, assay for transposase-accessible chromatin using sequencing (ATAC-seq), microscopy-based chromosome conformation capture (Hi-M) and single molecule localisation microscopy (SMLM) should to employed to characterise changes in chromatin compaction and 3D arrangement in response to changes in oxygen availability.

## Open Access

Open access for this article was enabled by the participation of the University of Liverpool in an all-inclusive *Read and Publish* pilot with Portland Press and the Biochemical Society under a transformative agreement with JISC.

## Abbreviations

2-OG: 2-oxoglutarate
ATAC-seq: assay for transposase-accessible chromatin using sequencing
ATM: A-T mutated
CBP: CREB-binding protein
CTAD: C-terminal transactivation domain
EHMT2: euchromatic histone lysine methyltransferase 2
FIH: factor inhibiting HIF1
HATs: histone acetyl transferases
HDACs: histone deacetylases
HIF: hypoxia-inducible factor
Hi-M: microscopy-based chromosome conformation capture
HMTs: histone methyltransferases
HP1BP3: heterochromatin protein 1 binding protein 3
JmjC: Jumonji-C
KAT5: lysine acetyltransferase 5
KDM: lysine-specific demethylase
MAT2A: methionine adenosyltransferase 2A
PHD: prolyl hydroxylase
SETDB1: SET domain bifurcated 1
SIN3A: SIN3 transcription regulator family member A
SMLM: single-molecule localisation microscopy
TET: ten-eleven translocation
VHL: von Hippel–Lindau tumor suppressor
